# Digital Gaming to Improve Adherence Among Adolescents and Young Adults Living With HIV: Mixed-Methods Study to Test Feasibility and Acceptability

**DOI:** 10.2196/10213

**Published:** 2018-10-15

**Authors:** Amanda D Castel, Saba Qasmieh, Daniel Greenberg, Nicole Ellenberger, Tyriesa Howard Howell, Caleb Griffith, Brittany C Wilbourn, Kavitha Ganesan, Nadia Hussein, Gabriel Ralte, Natella Rakhmanina

**Affiliations:** 1 Department of Epidemiology and Biostatistics Milken Institute School of Public Health George Washington University Washington, DC United States; 2 Media Rez, Inc Washington, DC United States; 3 Children's National Health System Washington, DC United States; 4 Center for Prevention Science School of Social Work Rutgers University New Brunswick, NJ United States; 5 Henry M Jackson Foundation for the Advancement of Military Medicine Bethesda, MD United States; 6 Walter Reed Army Institute of Research United States Military HIV Research Program Silver Spring, MD United States; 7 Department of Pediatrics School of Medicine and Health Sciences George Washington University Washington, DC United States; 8 Elizabeth Glaser Pediatric AIDS Foundation Washington, DC United States

**Keywords:** youth, HIV, adherence, video games, Wisepill, adolescents, digital technology, serious games

## Abstract

**Background:**

An estimated 50% of adolescents and young adults (AYA) living with HIV are failing to adhere to prescribed antiretroviral treatment (ART). Digital games are effective in chronic disease management; however, research on gaming to improve ART adherence among AYA is limited.

**Objective:**

We assessed the feasibility and acceptability of video gaming to improve AYA ART adherence.

**Methods:**

Focus group discussions and surveys were administered to health care providers and AYA aged 13 to 24 years living with HIV at a pediatric HIV program in Washington, DC. During focus group discussions, AYA viewed demonstrations of 3 game prototypes linked to portable Wisepill medication dispensers. Content analysis strategies and thematic coding were used to identify adherence themes and gaming acceptance and feasibility. Likert scale and descriptive statistics were used to summarize response frequencies.

**Results:**

Providers (n=10) identified common adherence barriers and strategies, including use of gaming analogies to improve AYA ART adherence. Providers supported exploration of digital gaming as an adherence intervention. In 6 focus group discussions, 12 AYA participants identified disclosure of HIV status and irregular daily schedules as major barriers to ART and use of alarms and pillboxes as reminders. Most AYA were very or somewhat likely to use the demonstrated game prototypes to help with ART adherence and desired challenging, individually tailored, user-friendly games with in-game incentives. Game prototypes were modified accordingly.

**Conclusions:**

AYA and their providers supported the use of digital games for ART adherence support. Individualization and in-game incentives were preferable and informed the design of an interactive technology-based adherence intervention among AYA living with HIV.

## Introduction

The goals of the US National HIV/AIDS Strategy include increased HIV testing, linkage to care, retention in care, and viral suppression for all persons living with HIV [1]. For an estimated 36,000 adolescents and young adults (AYA) aged 13 to 24 years living with HIV, these are particularly critical goals [2]. AYA accounted for 22% of all new HIV infections in 2015, and those AYA who were perinatally infected are aging into adulthood [3]. Furthermore, with treatment guidelines recommending early initiation of antiretroviral treatment (ART), AYA represent a significant population being prescribed daily ART medications [4]. Despite the high efficacy of new ART drugs and more available fixed-dose single tablet per day ART regimens, adherence to daily treatment remains suboptimal among AYA; less than half (47%) of youth aged 18 to 24 years living with HIV in 2016 were virologically suppressed [5].

As the chronic nature of HIV becomes apparent to youth, placing emphasis on adherence support is essential. As many as 50% of AYA or their caregivers report a failure to adhere to prescribed regimens [6-9], and a review of more than 50 studies on pediatric HIV infection confirmed that 42% to 80% of youth had suboptimal ART adherence [10]. AYA living with HIV are at particular risk of poor adherence due to specific challenges. They are experiencing a developmental period with increased peer pressure, risk-taking, and a desire to be similar to their HIV-negative peers [11]. Autonomy also differs between AYA and adults living with HIV, as younger persons must frequently rely on parents or caregivers for treatment [12,13]. Furthermore, AYA living with HIV may experience a higher frequency of cognitive deficits, depression, and substance abuse issues compared to general populations [14]. Therefore, the development of effective, youth-friendly interventions to improve adherence among AYA living with HIV is critical to improving adherence and treatment outcomes among this population.

Current approaches to ART adherence monitoring and support include text messaging, self-reporting, pill counts, pharmacy refill assessments, and electronic monitoring with devices such as Wisepill, a portable medication dispenser that emits a wireless signal when opened, used for monitoring real-time adherence [15-24]. While a few studies have provided evidence of the feasibility, acceptability, and performance of electronic monitoring of ART adherence, use of the Wisepill dispenser for real-time adherence monitoring is currently understudied; the dispenser appears to be a promising adherence strategy to explore. Digital game-based interventions also have the potential to be a promising approach to increasing treatment adherence among AYA. These approaches capitalize on the daily interests of AYA who are estimated to spend as many as 7 hours per day interacting with computers, cell phones, and video games [25,26].

Combining Wisepill technology and youths’ interest in digital gaming, we aimed to develop an intervention to link on-time Wisepill openings to in-game incentives to increase ART adherence among AYA living with HIV. As part of our gaming design process, we sought to better identify factors associated with nonadherence and identify digital gaming preferences among AYA and their health care providers. The objectives of this analysis were to use a formative research approach to describe barriers and facilitators to ART adherence among AYA living with HIV, identify their preferred digital game characteristics, and assess the feasibility and acceptability of the proposed intervention.

## Methods

### Study Setting and Approach

The study was conducted by a multidisciplinary research team comprising epidemiologists, qualitative researchers, HIV clinicians, and game development experts in Washington, DC. All study materials were reviewed and approved by the George Washington University and Children’s National Medical Center (CNMC) institutional review boards. Participants were recruited from CNMC Special Immunology Services (SIS), a clinic that provides care for HIV-exposed and infected infants, children, and adolescents in the DC metropolitan region. The SIS primarily serves perinatally and behaviorally infected patients from birth to 24 years old and provides a range of HIV specialty services including medical care, case management, subspecialty referrals, mental health services, and nutritional support. These services are provided by an experienced team of physicians, nurses, social workers, psychologists, and clinical and behavioral researchers.

The formative portion of this study included 3 phases: phase I, game prototype development and subsequent game revisions; phase II, a focus group discussion (FGD) with treatment providers of AYA; and phase III, a series of FGDs among AYA ages 13 to 24 years living with HIV. In phases II and III, each FGD was conducted by 2 moderators: (1) a clinician researcher to facilitate discussions regarding HIV knowledge and adherence and (2) the videogame developer to demonstrate the game prototypes and lead discussions on game design and development.

### Phase I: Game Prototype Development and Revisions

#### Theoretical Framework

**Figure 1:**
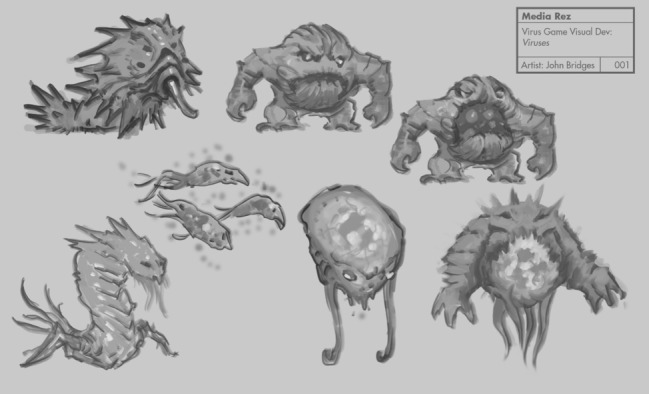
Virus Fighter images include HIV-like monsters that would invade CD4 cells.

Previous games for behavior change have shown positive results by using the compelling nature of game play to make health education entertaining and shift attitudes and emotions about chronic illness and treatments [27,28]. In our gaming design, we sought to use Wisepill dispensers for real-time adherence measurement and as a mechanism to provide incentives (eg, power-ups) for game play through the granting of microtransaction points for on-time pill box openings. Guided by Bandura’s social cognitive theory [29], a widely used framework that can be used to assess HIV adherence [30-32], we hypothesized that our intervention would positively influence self-efficacy, risk perceptions, knowledge of HIV treatment goals, and social support of study participants. Using this framework, iterations of game prototypes were developed by Media Rez including a project code-named Virus Fighter/Body Voyage, designed to teach players about the effects of HIV on the body and immune system, including important concepts such as poor adherence and medication resistance (Figure 1).

**Figure 2:**
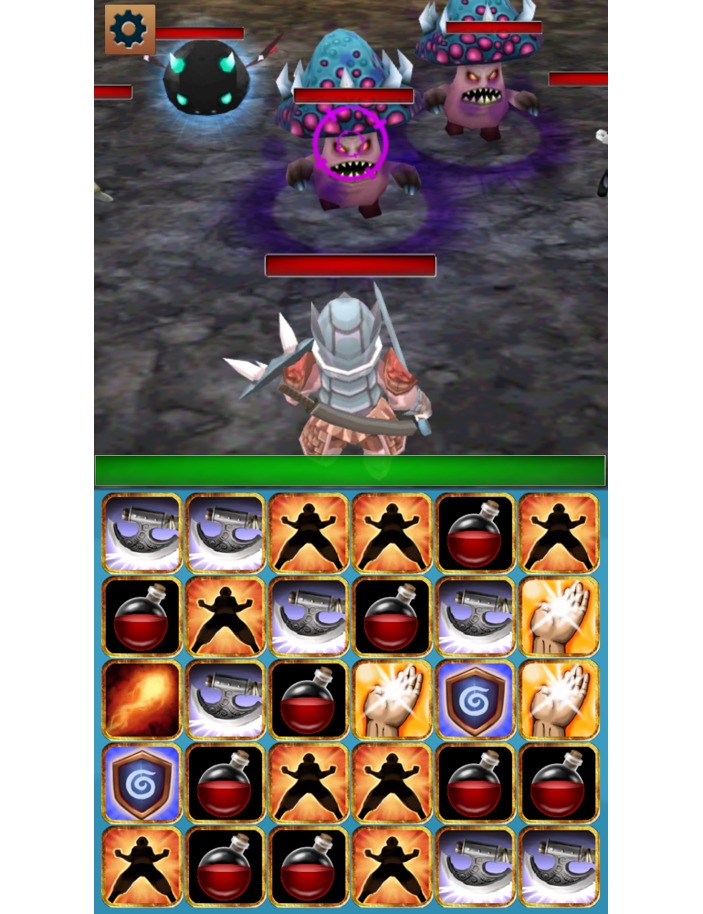
In the Adherence Warrior game, players act as heroes protecting their village from invading monsters. To attack the monsters, three or more tiles need to be matched in a row. Players need energy to progress in the game, and they can gain this energy by opening their pillbox on time.

This prototype demonstrated through gaming how HIV would get stronger if the player did not use ART as a weapon against the virus while also reinforcing how HIV is weakened if the player used ART. A second game, Adherence Warrior, was also developed with no explicit intent to be educational that included features that HIV care providers could potentially use as teachable moments to help educate AYA about the importance of ART adherence (Figure 2).

#### Game Modifications

Participant feedback from both health care providers and AYA was analyzed and informed revisions made to the initial game prototypes. This feedback was used to determine the optimal set of game features for the game prototypes used in a subsequent pilot intervention. As a result of the AYA feedback specifically, production on the Virus Fighter/Body Voyage game was suspended. In order to create sufficient game content to engage a variety of participants, prototypes of 3 distinctly different Wisepill-connected games were developed based on FGDs and survey feedback regarding desired game features. Participants would be able to play any of the 3 games at their discretion: a modified version of Adherence Warrior, the Berry Game, and Cat O’Polt, a new game that was developed in order to provide a game that was of moderate difficulty, potentially interesting to AYA, and adventurous. The games and pillbox interactions were also designed to meet important privacy, motivation, and information goals (Table 1).

#### Adherence Warrior Game

Adherence Warrior (Figure 2) features heroes in a fantasy world protecting their home village from invading monsters. The player manages a group of 3 brave adventurers who use weapons and magic to stop the invading monsters. To attack the monsters, 3 or more tiles are required to be matched in a row. Tiles can be moved 3 times for each of the 3 characters, which allows 9 attempts to make a match before the monster’s attack. Players can collect many new kinds of tiles resulting in new kinds of magic spells, skills, attacks, and healing. Players also need energy to progress in the game, and unlike in similar games, players cannot gain this energy by in-app purchases; they can earn it through adherence. Players can figure out unique vulnerabilities in different monsters and use the most effective tiles against those weaknesses.

**Table 1:** Features of final games.

Feature	Description	Function
Privacy	Making games about players fighting HIV conveys a risk of unintentionally revealing a patient’s HIV status when they would prefer privacy. To prevent this risk, we changed plans for a Body Voyage game fighting viruses and made 3 games designed to protect patient privacy and minimize the risk that using the intervention would disclose their status.	Increases app engagement by meeting the privacy needs of adolescent and young adult HIV patients.
Motivation	By making a direct connection between daily adherence and the kind of daily, in-game power-ups that players usually have to pay for with microtransactions, we attempted to motivate patients to take their medication on time. Opportunities to earn greater in-game power and access increases motivation for both engagement with the games and adherence with antiretroviral treatment (ART).	Direct motivation to increase adherence.
Motivation	The game incentivizes cooperation by letting the player build up available healing tiles and defensive tiles by not using them on less-injured heroes but instead saving them up for bigger healing effects on heroes who are more injured. In this way, the game puts the player in a caregiver role.	Having player in caregiver role motivates patients in their current self-care role to adhere to their ART.
Information	The system knows when patients do not open their pillboxes on time and sends out a feedback reminder when this happens in the form of a text message (which does not disclose anything about HIV).	Providing this timely information about adherence affirms adherence behavior and serves as a cue to action in a way that does not breach privacy.

Although the game contains no explicit HIV education (to avoid violating patient privacy), it was designed to have teachable moments that HIV care providers could potentially use to explain complex concepts associated with treatment. Provider FGDs reported that one of the most difficult concepts to teach is the potential for viral resistance to ART, which is commonly taught by metaphor. By creating the vulnerability system in the game, the provider can emphasize this similarity with HIV resistance. This provides a conceptual approach to understand complex adherence concepts and behaviors. In this way, the game not only avoids breaching the privacy of AYA living with HIV but also avoids coming across as didactic. The game itself does not try to lecture the patient through onscreen text but provides experiences designed to be used as teachable moments by caregivers. Enabling educational opportunities and points of connection for providers and patients through the game could be more effective for behavior change than simply having the game display health messages that are often ignored.

#### Berry Match

**Figure 3:**
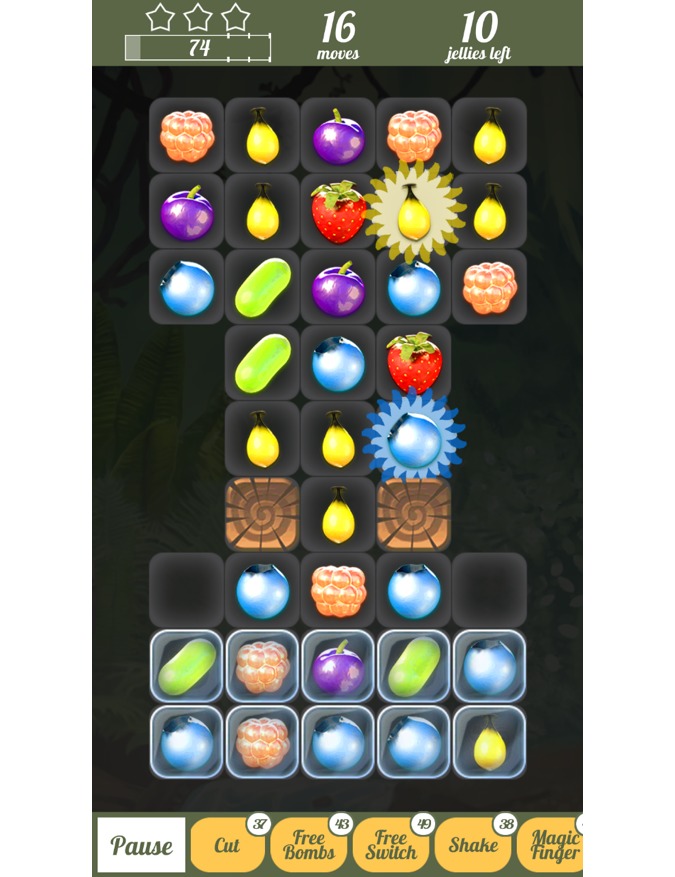
In the Berry Match game, players try to match as many berries as possible. Points are awarded for all the berries eaten. Players receive in-game currency in the form of special power-ups by opening their pillbox on time.

The objective of the Berry Match (Figure 3) game is to match as many identical berries as possible.

If 3 or more berries are matched in a row, the berries will switch places and the 3 matching berries will disappear and new berries descend. Points are awarded for all berries “eaten.” Some levels have limited amounts of moves to win while others can be beaten by reaching the target score. Special blocking and trapping tiles add to the challenge. When the player takes his or her medication on time, they receive in-game currency that can be spent on special power-ups to solve especially tricky levels of the game. In similar commercial games, players must purchase these power-ups with cash through in-app purchases; in our gaming design they can earn the in-game currency by opening their Wisepill boxes on time.

#### Cat O’Polt

**Figure 4:**
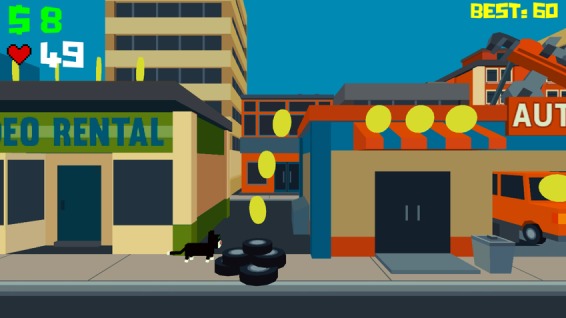
In the Cat O'Polt game, players control a cat travelling through the city trying to collect coins while avoiding obstacles. The objective is to achieve the highest score possible before losing all of one’s gameplay lives. The player gains new lives by opening their pillbox on time.

The goal of Cat O’Polt (Figure 4) is to direct a cat as it travels through the city collecting as many coins as possible while simultaneously avoiding obstacles.

With the right timing, the cat can make huge jumps over obstacles and even vault over buildings. The objective is to achieve the highest score possible before losing game play lives. In our gaming design, players can gain new lives by opening their pillbox on time.

As the next phase of the study, the Wisepill dispenser was integrated within each game and was linked to in-game power-ups for daily Wisepill openings as an incentive to improve ART adherence.

### Phase II: Focus Group Discussion With HIV Providers

A semistructured FGD using a single category design was conducted among the specialty HIV care provider team at CNMC SIS and was aimed at identifying key facilitators and barriers to ART adherence among AYA and eliciting feedback on the game prototype and use of the Wisepill dispenser. The semistructured format allowed FGD moderators to probe emergent topics and themes during the discussion. Prior to the FGD, providers were administered a brief survey to collect basic demographic and practice-related characteristics. Providers were not remunerated for their participation in the FGD.

### Phase III: Focus Group Discussions With Adolescents and Young Adults

AYA ages 13 to 24 years living with HIV and disclosed as to their HIV status were recruited during routine clinic visits at CNMC SIS. A series of multiple-category FGDs were stratified by age (13 to 17 and 18 to 24 years) to account for developmental differences and potential variances in game characteristic preferences among participants. A total of 12 AYA participated in 6 FGDs; 3 FGDs among participants aged 13 to 17 years and 3 FGDs among participants aged 18 to 24 years. Participant feedback from the first FGD was analyzed, and revisions were made to the initial game prototypes. During the second stage of FGDs, participants from the initial FGDs as well as AYA who had not previously viewed the games were invited to participate.

Participants had the flexibility to explore multiple games and provide unrestricted input regarding user interface performance, acceptability of the art and animation, and response to game play. Additional input was also provided regarding the feasibility and acceptability of the games with respect to their potential to improve ART adherence.

Before the start of each FGD, participants completed a survey that collected information on their demographics, ART regimens, and current video game use. A separate survey using Likert scale ratings was administered to participants after demonstration of the video game prototypes to quantify their impressions of the games and their potential to improve adherence. All AYA participants were remunerated with gift cards and transportation vouchers for their participation in each FGD.

### Data Collection and Analysis

All FGDs were audio-recorded and professionally transcribed verbatim for data analysis. A systematic analysis guided by a constant comparative analytic framework was used to employ content analysis strategies through the use of thematic coding to determine major findings. Constant comparative frameworks objectively identify patterns in data and discover relations between ideas and concepts [33]. This framework supported the use of summative content analysis, which involves comparing the content of data to interpret the underlying context [34]. Content analysis was achieved through thematic coding, which links the common themes and ideas discussed among study participants [35]. Qualitative data analysis was organized using both NVivo version 9 (QSR International) and ATLAS.ti version 7 (Scientific Software Development GmbH) qualitative data analysis software by 2 independent coders and included 2 cycles: (1) each interview transcript was objectively analyzed using the aforementioned analytic approach and (2) coding across FGDs was compared to produce overall themes that support the purpose of the study. Codes were reviewed, and intercoder agreement was achieved where there were discrepancies in the categorization of the codes. Descriptive statistics were used to summarize response frequencies from the surveys. Mann-Whitney *U* tests were used to compare median responses to survey questions across 2 AYA age groups (13 to 17 years and 18 to 24 years).

## Results

### Provider Focus Groups

#### Provider Demographics and Focus Group Themes

The HIV provider FGD (n=10) included physicians (n=2), case managers (n=3), research coordinators (n=3), a clinic nurse (n=1), and a psychologist (n=1). The majority (6/10, 60%) of providers had at least 5 years’ experience prescribing ART and delivering treatment and adherence support services, conducting clinical research, and providing HIV counseling and testing to AYA. During the FGD, several recurrent themes emerged related to ART and the potential role of video games to improve adherence, including (1) patient understanding (or lack thereof) of the concept of adherence, (2) daily barriers to patient adherence, (3) facilitators and promotion strategies for ART adherence, (4) provider educational methods to educate and support HIV treatment, (5) perceptions of the role of video games in ART adherence support, and (6) desired characteristics of video games for the patients (Textbox 1).

**Summary themes identified from the HIV provider focus group discussions and representative quotes.**

Patient understanding of adherence and resistance

- “The concept of resistance...I don’t think people understand what it means to be resistant. We keep trying to explain [you know what] it means the drugs don’t work anymore...people do not see the consequence...”
- “...it’s associated with negativity as opposed to being something that’s empowering that I can take control of my medicines, I can take control of my life, I can have control over...”

Barriers to adherence

- “A lot of the times the patient...may have, like, issues about disappointing the clinicians and the providers and so they’ll be less forthcoming about their actual adherence, and then when you confront them about their viral load and why what they’re saying isn’t matching then they’ll, you know, give the puppy dog eyes and kind of disclose that ‘actually I haven’t been taking my medicine.’”
- “They don’t feel sick, they don’t feel like they need to take medicines, it’s very abstract in terms of me talking about ‘this is your CD4 count, this is your viral load, you take your medicine so your viral load goes down and your CD4 comes up’...but they don’t feel the importance of it...”
- “And I think that taking their meds a lot of what I hear is ‘Oh when I take my meds that reminds me that I have HIV. If I am reminded about HIV I just don’t want to deal with it.’ And as they get older and older it becomes more of an issue and they don’t see the consequences. They don’t see anybody dying around them from HIV, they don’t see the ramifications of it.”

Facilitators and promotion strategies

- “Actually our kids who are very concrete thinkers tend to do really well that they just take the medicine; they don’t think about all the ramifications of why I’m taking medicines and I’m going to have to do this for the rest of my life...”
- “I feel like those kids [who have been told their HIV-positive status sooner] do better than the ones who are 13 or 14 and it’s been a secret hidden and then they’re being told that [they have HIV]...Those are the ones that really seem to struggle [with medication adherence].”
- “...we do have a few kids though, very few, but we have a few kids where the opposite happens where they get old enough to take their own meds and they actually weren’t taking them and they do better and they all of the sudden become undetectable. When it was the parents’ responsibility it didn’t happen.”

Provider educational methods

- “So the way I’ve used it...gaming analogies, I’ve told ‘you need the grenade, you need the pistol and you need the AK-47, you need all 3 to kill off the virus. You can’t just do one or else it’s not going to work it’s going to get stronger...like you need all 3 you don’t need just one, it’s not going to work...’”
- “Red, green, and yellow light and it’s in regards to the immune system...I will tell patients frequently you’re now in the yellow zone that means that I’m seeing the decline in your immune system and I’m trying to urge you not to let it become red zoned. And then obviously there [are] some patients when I will say... we are in the red zone your immune system is weak and it’s not recovering. The longer we see it in the red zone the harder it will be to recover the immune system.’”
- “Because I’ve actually tried the video game analogy many times in terms of ‘you know those little power packs you have to get so that your energy level goes really high that’s what you’re trying to do with your medicines, get that power pack...’”

Initial perceptions of role of video games

- “Yes that incentive is there and yes that reminder is there but after a while it just becomes like ‘oh this is nothing I don’t really care, whatever.’”
- “I wonder, first of all I think about gender differences, I wonder if it’s going to be girls versus boys. And I think boys might be more interested in doing it.”
- “I don’t know how that’s going to help because I think some of our kids forget but I don’t think a lot of them forget. I think the thing rings and they go, ‘oh this thing’s ringing,’ and they continue with playing the video game...Maybe if the video game dispensed it...”

Desired characteristics of a video game

- “One thing that I think is really important is that the game not peg them as being HIV positive”
- “And it might send you a message saying you’ve been doing great...and the second reward is missing and this is what you could do if you want.”
- “And also I just want to say, to me, like, with the teenager is a very change in milieu. So I think having the same system of awards is boring. So I think what needs to be in any game is you need to completely change the system once they get it.”

#### Perception and Experience With Antiretroviral Treatment Adherence

Providers expressed difficulty conveying the importance of medication adherence and explaining the consequences of poor adherence (ie, resistance) to AYA. Providers emphasized that AYA usually do not want to disappoint providers and described AYA as having a desire not to “feel sick,” and not wanting to be reminded of having the virus. Providers also identified increasing difficulty with adherence as the patients got older—in addition to the perception among AYA that they were invincible. Providers emphasized the majority of AYA in care did not witness people dying from HIV and therefore did not perceive the long-term consequences of not taking the medications as a threat to their lives. Providers described characteristics of AYA living with HIV that made adherence challenging, including concrete thinking, being less organized, and lacking the maturity to control their own ART regimens. Providers also believed that learning one’s HIV-positive status at a young age was associated with improved ART adherence. Providers also described their efforts to use age-appropriate, culturally relevant analogies to increase understanding of viral resistance and ART adherence among AYA in care.

#### Perspective on the Potential Role of Video Games

With regard to the feasibility and acceptability of using video games to improve adherence, many providers were initially skeptical about the ability of gaming to improve adherence. They suggested that once the novelty of the gaming wore off, adherence levels might decrease. Providers also expressed concern that gender differences could emerge with males potentially being more engaged in gaming compared to females. Despite providers’ initial skepticism, after seeing and experiencing the game prototype, the majority of providers expressed optimism and provided specific feedback for improving game design. Overall, providers were supportive of exploring the proposed gaming intervention and recognized its potential positive role in improving adherence among AYA in care.

### Focus Group Discussions With Adolescents and Young Adults

#### Demographics

**Figure 5:**
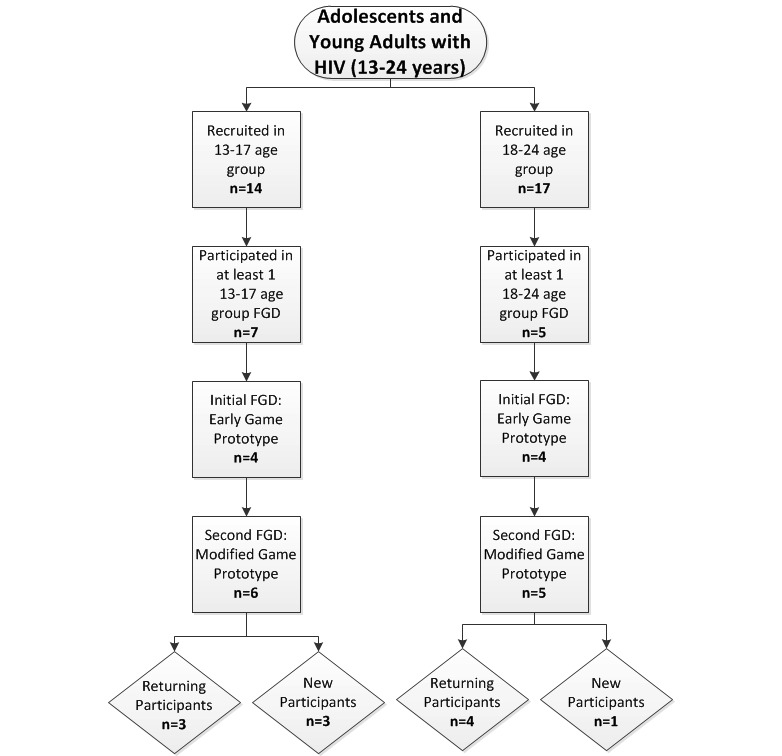
HIV-infected adolescent and young adult focus group participant recruitment. FGD: focus group discussion.

A total of 31 AYA were recruited for the study; however, only 12 were able to attend and participate in the FGDs. Among the 12 AYA who participated in the 2 rounds of FGDs, 7 were aged 13 to 17 years old and 5 were aged 18 to 24 years old (Figure 5).

**Table 2:** Adolescent and young adult demographic, disclosure status, adherence, and access to video games characteristics (N=12).

Characteristic^a^		13-17 years (n=7)	18-24 years (n=5)	Total (N=12)
Age (years), mean (range)		14.7 (14-16)	19.2 (18-22)	16.6 (14-22)
**Sex, n (%)**					
	Female	4 (57)	3 (60)	7 (52)
	Male	3 (43)	2 (40)	5 (42)
**Race/ethnicity, n (%)**				
	Non-Hispanic black	7 (100)	4 (80)	11 (92)
	Other	0	1 (20)	1 (8)
**State of residence, n (%)**				
	Maryland	6 (86)	2 (40)	8 (67)
	District of Columbia	1 (14)	3 (60)	4 (33)
**Education level, n (%)**				
	Grades 1-8	4 (57)	0	4 (33)
	Grades 9-11	3 (43)	1 (40)	4 (33)
	High school graduate/general equivalency diploma	0	4 (80)	4 (33)
**Time since HIV disclosure prior to study enrollment, n (%)**				
	<6 months	2 (29)	0	2 (17)
	6 months to 5 years	2 (29)	0	2 (17)
	>5 years	3 (43)	5 (100)	8 (67)
**Storage of HIV medications, n (%)**				
	In my bedroom	3 (43)	4 (80)	7 (58)
	In the kitchen	4 (57)	1 (20)	5 (42)
Pills per day, mean (range)		4.3 (1-8)	2.2 (1-4)	3.4 (1-8)
**Medication regimen, n (%)**				
	Once per day	4 (57)	5 (100)	9 (75)
	Twice per day	2 (29)	0	2 (17)
	≥3 times per day	1 (14)	0	1 (8)
**Pillbox/dispenser use, n (%)**				
	In the past	1 (14)	1 (20)	2 (17)
	Currently	4 (57)	3 (60)	7 (58)
	Never	2 (29)	1 (20)	3 (25)
**Adherence strategies^b^, n (%)**				
	I do it myself	3 (43)	2 (40)	5 (42)
	A relative helps me remember	5 (1)	2 (40)	7 (58)
	I set an alarm	0	2 (40)	2 (17)
	I receive a text reminder	0	1 (20)	1 (8)
**7-day adherence, n (%)**				
	All the time (100%)	4 (57)	1 (20)	5 (42)
	Most of the time (51-99%)	3 (43)	4 (80)	7 (58)
	Half of the time to never (0-50%)	0	0	0
Video game device in household, n (%)		7 (100)	5 (100)	12 (100)
**Gaming devices in household^b^, n (%)**				
	Wii	5 (71)	2 (40)	7 (58)
	Xbox	4 (57)	2 (40)	6 (50)
	Nintendo	0	3 (60)	3 (25)
	Computer	2 (29)	2 (40)	4 (33)
**Frequency of game play, n (%)**				
	Daily	0	1 (20)	1 (9)
	Only on weekends	2 (29)	0	2 (18)
	Anytime	2 (29)	4 (80)	6 (54)

^a^Totals may not sum to 100% due to missing data.

^b^Participants were allowed to check all that applied.

The mean age of participants was 16.6 years, 92% (11/12) were black, and 58% (7/12) were female (Table 2). All participants had been told about their HIV status, and many participants (8/12, 67%) had known for more than 5 years. All participants were prescribed ART, and 75% (9/12) were on a once-a-day ART regimen. Younger adolescents (ages 13 to 17 years) had a higher pill burden compared to older AYA (4.3 vs 2.2 mean number of pills, respectively). Seven participants (58%) were currently using some type of pillbox at the time of the FGDs.

#### Perspectives on Adherence

The following themes were identified during the AYA FGD: general HIV and ART knowledge, knowledge of viral resistance and ART adherence, barriers and facilitators to adherence, interest in and use of video games, and acceptability of video games for improving adherence (Textbox 2).

#### Knowledge of HIV, Adherence, and Barriers and Facilitators to Adherence

Participants in the younger FGD had a conceptual understanding of HIV, whereas older FGD participants demonstrated a deeper understanding of HIV and adherence concepts. Participants across both age groups expressed a similar understanding of viral resistance and adherence. When discussing barriers associated with adherence to ART, participants revealed a broad array of factors such as not wanting to be different from their peers, being afraid of inadvertently disclosing their status by taking the medications, having difficulties with the taste and size of medications, and facing time management challenges. Participants in all AYA FGDs shared personal motivating factors and facilitators associated with improving adherence.

#### Feasibility and Acceptability of Video Games for Adherence

All AYA participants had a video game device in their house with the most commonly reported gaming platforms being Wii (7/12, 58%) and Xbox (6/12, 50%). Additionally, 80% (4/5) of older AYA reported unrestricted access to video games compared to 29% (2/7) of younger AYA (Table 2). Prior to introducing the initial video game prototype, participants were asked to describe enjoyable aspects of video games and motivating factors for playing games. Participants generally expressed that they liked games that were engaging, had intense adventure, and posed a challenge. Discussion about the use and frequency of video game playing indicated that length of time spent on video game playing was dependent on the challenging nature of the game. Participants in the younger age group reported enjoying games as a leisure activity*,* while participants in the older age group were more interested in challenging games.

During the first round of FGDs, participants viewed demonstrations of the Wisepill ART dispensers, artwork and images for the prototype of Virus Fighter/Body Voyage (Figure 1), a game about the immune system, and an initial version of the Adherence Warrior (Figure 2) game. All FGD participants were comfortable with the Wisepill dispensers and believed that their medications would fit and that they would be able to carry the dispenser around without any issues. When introduced to the concept of linking the Wisepill to in-game incentives to improve medication adherence, AYA demonstrated acceptance of the concept. Younger participants thought the Wisepill would serve as an additional reminder to help them remember to take their medications, whereas the older participants focused more on the game content and emphasized a desire for the games to be engaging and interactive.

**Summary themes identified from the HIV-infected youth and young adult focus group discussions and representative quotes.**

**General HIV/antiretroviral treatmen knowledge**

How antiretroviral therapy works and how it affects the body

- “I mean I heard it before, I forgot. We used to talk about it a lot but I wasn’t listening ‘cause I didn’t understand. ‘Cause I didn’t want to take my medicine.” [13 to 17 years]
- “My viral load goes down by taking the meds...taking the meds every day.” [13 to 17 years]
- “I guess if you don’t take it [ART] for a long period of time then the virus can start, like, making your body feel weird. If you don’t start taking your medicine. You might, like, feel weaker, or...Medicines will stop being effective. ‘Cause the virus will, I guess it’ll get used to it. Or, something like that.” [13 to 17 years]

Antiretroviral therapy and HIV knowledge

- “I just know that you get born with it or you get it through like sex and stuff. And it’s like a disease that’s in your immune system that takes over your immune system.” [18 to 24 years]
- “You want the viral load to be really low because that’s how much the virus is in your body, you want it to be low.” [18 to 24 years]
- “They got some [pills] out here that’s one a day, they got like, probably like 2 or 3 different kind that’s one a day and then they got other kinds that’s not one a day but for the people who take the ones that’s not one a day it’s probably because when they were younger, like if they had to live with it all their life like me then it messed up their medicine at first then now we can’t take the one a day pill.” [18 to 24 years]

**Knowledge of adherence and resistance**

Impact of not taking meds daily

- “It’s like when you stop taking your medicine and it stops working or you want to take it and it doesn’t work.” [13 to 17 years]
- “It’s when your medicine don’t work, right, or don’t want to...like help your body...I mean ‘cause it doesn’t work anymore.” [13 to 17 years]
- “If you take it for a long time period that regardless. I don’t know...medicines stop being effective...” [13 to 17 years]
- “Resistance is if I’m suppose[d] to take 4 pills a day, right, so I stop taking those 4 pills or something like that or maybe I might just take 3 of the 4 pills and that 1 pill or those 4 pills that I’m supposed to be taking that will eventually help me to, you know, like [new] medicine and stuff like that it’s not going to work because I didn’t take it and I was playing around with it.” [18 to 24 years]
- “I think I heard it before but now I really remember what they were saying...” [18 to 24 years]
- “Yea, I’ve heard of resistance...resistance to medication...so like if you take it [medicine] and then if you don’t take it for a long time and then take it again you could be resistant to it and then it won’t work no more.” [18 to 24 years]

**Barriers/facilitators to adherence**

Not wanting to be different, motivation from family, and forgetfulness

- “I always have been taking my medicine. But yes, I did have a time when I didn’t [take my medicine]. Nobody else was taking medicine and I felt like I was the only one who was and I didn’t want to be different and I didn’t want to be the only one who was taking medicine and then questions of why I am taking it...” [13 to 17 years]
- “I felt I need to be around to see my brother graduate and I want to still be around to talk to people, motivation...” [13 to 17 years]
- “I can remember taking it in the morning but sometimes after school I forget because I’m doing like my homework and stuff.” [13 to 17 years

Difficulty swallowing, reminders, and forgetfulness

- “It’s, um, it was just hard taking it ‘cause the pill was nasty or too big to swallow and the liquid was just disgusting...” [18 to 24 years]
- “I set 3 alarms every hour and I put those 3 alarms on repeat 3 times.” [18 to 24 years]
- “When I turned 15 and 16 going to high school and all that then you start forgetting because it was like a whole bunch of stuff on my plate you know at the time.” [18 to 24 years]

**Interest and use of video games**

Appeal of games

- “Just a good way to pass time. Especially if I’m not doing anything, like on Sundays. Just to get passed time I guess.” [13 to 17 years]
- “I like a lot of adventure...” [13 to 17 years]
- “I play after homework...but in the summer, my mom doesn’t care.” [13 to 17 years]
- “Well it keeps my attention but I like a game that has an interesting story behind it or just keeps me involved ‘cause I get a game quick.” [18 to 24 years]
- “Well I play all types of games but one game my head was stuck on and that’s because I can’t beat it because it keeps changing is War Frame.” [18 to 24 years]
- “...the harder the challenge the better...” [18 to 24 years]

**Acceptability of video games for adherence**

Perceptions regarding the potential of games to assist with adherence

- “Yeah...because I need something besides my alarm.” [13 to 17 years]
- “I think if you like playing video games you’d remember to take your medication.” [13 to 17 years]
- “I wish we could like have our own little profile.” [13 to 17 years]

Suggestions for improving the game prototype

- “I don’t know, it might work but for me there’d have to be a game where it’s like, kind of like how XX said, like a puzzle or something like that or like a challenge game or whatever...” [18 to 24 years]
- “Like I mean every person is different but for me it would be like a puzzle game. Like some of it makes you think and you have to like work for it at the end and then they’ll be like a message or something.” [18 to 24 years]
- “I like the power-ups because you get it to do different like variety of stuff so you get to choose different stuff...if you have different stuff you can earn a higher score.” [18 to 24 years]

In addition to the interactive FGDs, brief surveys using Likert scales were used to evaluate acceptability of the Wisepill and game prototypes among AYA (Table 3).

On a scale of 1 to 5, with 5 indicating strong agreement, most participants agreed the dispenser format was convenient (median 4.0) and linking it to the games would not be an issue (median 5.0). In assessing game design upon viewing the artwork for Virus Fighter, younger participants were less likely than older participants to like the idea of playing games about the immune system (median 4.0 vs 5.0, respectively; *P*=.01) and significantly more likely to agree that they preferred playing games about topics other than the immune system (median 4.0 vs 5.0, respectively; *P*=.01).

The feedback from the initial AYA FGDs led to suspension of production on the Virus Fighter/Body Voyage game, revisions of the Adherence Warrior game, and the development of a third game prototype, the Berry Game, both of which were shared with AYA during the second round of FGDs.

After playing the two intervention game prototypes, most participants were optimistic of the games’ abilities to help them improve their adherence and provided suggested revisions that were incorporated by the researchers. Among the suggested changes were allowing for the customization of characters including both male and female avatars, adding music, having an in-game notification or reminder that encourages players to take their medication, and increasing the diversity of the games. Participants conveyed interest in games with a bonus stage and power-up capabilities that enabled additional features leading to more points. Participants also favored power-ups as a reward for ART adherence over gaining additional points, which emphasized their preference for playing games for the challenge rather than for winning.

AYA reported that they thought the games were interesting (median 5) and satisfying (median 5). Participants in the 18- to 24-year age group were more likely to report that the games were fun (median 5.0 vs 4.0, *P*=.03) and to prefer games about subjects other than the immune system (median 5.0 vs 4.0, *P*=.01) (Table 3). Using a separate Likert scale with a range of 1 to 4 with 4 indicating high likelihood, participants reported that they would play the games if they were available to them (median 4.0), they would use the games to help with taking their HIV medications (median 4.0), and they would be more likely to take their medication if they could get better power-ups in the games (median 4.0). Overall, preferred game characteristics, regardless of age group, included being (1) challenging enough to keep one’s attention, (2) individually tailored based on interests, (3) competitive, (4) containing a virtual reward system to provide in-game incentives, and (5) easy to play.

**Table 3:** Acceptability of game prototype stratified by age group.

Game characteristics^a^	13-17 years (n=7), median response	18-24 years (n=5), median response	Total (N=12), median response
The game playing was very interesting^a^	5	5	5
The game playing was satisfying^a^	4	5	5
The game playing was fun^a^	4^c^	5^c^	5^c^
It was easy to learn how to play the game^a^	4	4	4
I liked the art and animation^a^	4	5	4
I liked the game environment^a^	3.5	4	4
I liked the game interface^a^	4	4	4
I like the 7-day organizer format of the pill dispenser^a^	4.5	5	5
The link to the pill dispenser was not a problem for me^a^	5	3	4
I like the idea of playing games about the immune system fighting off invaders^a^	4	5	5
I like the idea of playing games about subjects other than the immune system^a^	4	5	4
Games about the immune system make me more interested in the subject^a^	3.5	5	4
I would like to get a pop-up reminder to take my medications from the game on my phone^a^	4.5	4	4
I would play these games if I had them^b^	4	3	4
I would recommend the games to a friend^b^	3	3	3
I would be interested in playing these games in multiplayer mode with my friends^b^	3	2	2
I would use these games to help with taking my medications^b^	4	3.5	4
I would be more likely to take my medication if it would get me more points in the games^b^	3	3	3
I would be more likely to take my medications if it would get me better power-ups in the games^b^	4	4	4

^a^Responses provided on a scale of 1 to 5: *strongly agree* (5), *somewhat agree* (4), *neither agree nor disagree* (3), *disagree* (2), *strongly disagree* (1).

^b^Responses provided on a scale of 1 to 4: *very likely* (4), *somewhat likely* (3), *not very likely* (2), *don't know* (1).

^c^Significant differences between age groups using *t* tests (*P*<.05).

## Discussion

### Principal Findings

This study, which explored the potential for using gaming technology to support long-term treatment of HIV among AYA, demonstrated that it would be feasible and acceptable to use a digital gaming intervention linked to Wisepill dispensers to improve ART adherence. Qualitative data collected through FGDs elicited information from both HIV care providers and AYA participants. The FGDs with HIV care providers revealed concerns regarding the level of understanding of the importance of adherence and viral resistance as well as age-related challenges such as nondisclosure, treatment fatigue, and limited capacity to foresee long-term consequences of nonadherence among their AYA patients. The challenges to daily ART adherence were echoed by the AYA FGD participants as they identified common barriers to treatment adherence and strategies to improve adherence.

Despite the providers’ concerns, participating AYA demonstrated an understanding of the concepts of resistance and adherence and acknowledged the multiple adherence barriers they face, which included changes in daily routines and fear and anxiety of disclosing their status to others, all of which are consistent with the existing literature [11,36,37]. Our discussions also indicated that AYA considered that having an understanding of the chronicity of HIV and the ability to set long-term goals were facilitators of adherence. These findings are not surprising considering the complex developmental periods of adolescence and young adulthood of altered levels of identification, self-regulation, and influence from peers [11]. As adolescents mature into young adults, strategies and messages to support adherence, self-management, and well-being should reflect shifts from concrete to abstract thinking and from an invulnerable to self-preserving mindset [12]. This is reinforced by our finding of AYA expressing willingness to try more creative approaches to facilitate better medication adherence, including playing video games.

The proposed approach of using Wisepill dispensers linked to games with microtransactions and power-ups was affirmed as acceptable by both HIV care providers and AYA living with HIV. Importantly, AYA participants in our study were receptive to the concept of using Wisepill dispensers for their adherence monitoring as the majority of them had some past experience with different types of pillbox or pill dispenser use. The feasibility and acceptability of using wireless electronic device monitors for adherence among HIV-infected persons has been demonstrated in other settings including in China and Uganda. In Uganda, adult HIV-infected patients described the Wisepill device easy to use and convenient, and it was found to be noninferior to the Medication Event Monitoring System pill bottle caps [15]. Further, among Chinese children aged 10 to 15 years, Wisepill dispensers were reported to be an acceptable and rigorous method of evaluating ART adherence and were found to be potentially useful for adherence support among adolescents living with HIV [38].

Our study was also informative in gathering key information from game users and providers about the role and the design of the game within the context of health promotion. All the AYA participants reported regular technology use and unfettered access to video games and provided overall positive feedback on the proposed game prototypes. Interestingly, among HIV care providers, initial discussions regarding the potential for gaming to improve adherence indicated some hesitation and skepticism, although some of them had described previously using gaming analogies to educate patients about HIV and adherence. Even though some providers used gaming analogies to explain the role of adherence in treatment, they were clear that the games should avoid defining the youth as being HIV-infected and should instead be positive and motivational. Similarly, the AYA participants were not particularly interested in an educational or HIV-themed game. This desire for noncontent-related games among providers and AYA in our study is in contrast to some other games developed for health-related purposes, which are both educational and disease-specific [39-41]. We believe that the desire to avoid the direct topic of HIV in gaming is closely linked to the sensitive nature of HIV disease and external and internal stigma experienced by people living with HIV. Therefore, our games were not specifically HIV-related nor did they include a community-gaming aspect to intentionally avoid inadvertent disclosure. In future iterations of the game, it may be possible to incorporate a community-gaming aspect in a confidential manner as well as potentially link the Wisepill device to existing popular games to address concerns about the durability and sustained use of a particular set of games.

Through our iterative study design, AYA provided important feedback and concrete suggestions to the game development process. They preferred games that were challenging, individually tailored, included a virtual reward system, and were easy to play. In other studies, video games that were developed using input from their target population have been proven effective in the chronic disease management of health conditions in AYA such as asthma, cancer, and diabetes [42]. In fact, a video gaming approach has recently been tested among AYA living with HIV that incorporates an adherence-based app inclusive of games [39]. The developers reported that AYA enjoy the interactive and customizable app elements and found this approach to be highly acceptable [39]. Further, studies among youth with chronic diseases have also found that game interventions result in improved self-management and decreased unanticipated medical visits [43]. Based on these studies and our data, we anticipate that our versions of the games will appeal to AYA living with HIV and will be effective in supporting medication adherence and improving HIV outcomes such as viral suppression.

### Limitations

There are several limitations to our study worth noting. Despite high numbers of initial youth recruited, the number of youth participating in the FGDs was relatively small and participants were drawn from a convenience sample recruited from a single clinical center. Although common in qualitative research, the small sample size limits the generalizability of the study findings. Further, all participants identified as black or African American, reflecting the high burden of infection among these populations in the DC region as well as in the United States [28]. While this may limit generalizability of findings to AYA of other races or ethnicities, it should be noted that none of the video game prototypes were uniquely designed for black youth and were instead based on common video game prototypes used among diverse populations. Additionally, FGD moderators can affect the outcome of an FGD by intentionally or inadvertently inserting their personal biases into the participants’ exchange of ideas. Since one of the moderators of our FGD was the developer of the video games being tested, the FGD participants could have been led into reaching certain assumptions and may have been somewhat influenced in their opinions. We attempted to reduce this potential bias by including a clinician-researcher as a moderator in each FGD to deter biased discussion and responses.

### Conclusions

AYA living with HIV face multiple challenges with medication adherence and must develop effective strategies to help them remember to take their medications. Similarly, HIV care providers strive to support ART adherence and educate AYA about the long-term consequences of nonadherence. Given these challenges, the development of effective, youth-friendly interventions to improve adherence among AYA living with HIV is critical to achieving better health and outcomes of HIV disease. In the era of the widespread use and availability of technology including mobile phones and video games, the concept of using a technological approach to improve medication adherence is supported by both AYA and their HIV care providers. Inviting buy-in from youth to elicit their preferences as end-users of the game-based intervention and modifying the games accordingly to include non-HIV focused, interactive, adventurous, and challenging games is likely to result in stronger interest and engagement in the future intervention. The potential effectiveness of this approach will be tested in a future study among a cohort of 13- to 24-year-old HIV-infected AYA with suboptimal ART adherence at CNMC.

## Abbreviations

ART: antiretroviral treatment
AYA: adolescent and young adult
CNMC: Children’s National Medical Center
FGD: focus group discussion
SIS: Special Immunology Services
